# Malignant tumor is the greatest risk factor for pulmonary embolism in hospitalized patients: a single-center study

**DOI:** 10.1186/s12959-021-00334-2

**Published:** 2021-10-30

**Authors:** Kaoru Fujieda, Akiko Nozue, Akie Watanabe, Keiko Shi, Hiroya Itagaki, Yoshihiko Hosokawa, Keiko Nishida, Nobutaka Tasaka, Toyomi Satoh, Ken Nishide

**Affiliations:** 1 Tsukuba Medical Center Hospital, Tsukuba, Ibaraki, Japan; 2 Department of Obstetrics and Gynecology, Faculty of Medicine, University of Tsukuba, 1-1-1 Tennoudai, Tsukuba, Ibaraki, 305-8575 Japan

**Keywords:** Venous thromboembolism, Pulmonary embolism, Gynecological cancer

## Abstract

**Background:**

This study aimed to investigate the background of patients who presented with pulmonary embolism (PE) on contrast-enhanced chest computed tomography (CT) and to explore the risk factors for PE.

**Methods:**

This study included a review of the medical records of all 50,621 patients who were admitted to one community hospital between January 1, 2013 and December 31, 2017. Data on sex, age, risk factors related to blood flow stagnation (obesity, long-term bed rest, cardiopulmonary disease, cast fixation, long-term sitting), risk factors related to vascular endothelial disorder (surgery, trauma/fracture, central venous catheterization, catheter tests/treatments, vasculitis, antiphospholipid antibody syndrome, history of venous thromboembolism (VTE)), and risk factors related to hypercoagulability (malignant tumor, use of oral contraceptives/low-dose estrogen progestin/steroids, infection, inflammatory enteric disease, polycythemia, protein C or protein S deficiency, dehydration) were evaluated.

**Results:**

Of all inpatients, 179(0.35%) out of 50,621 were diagnosed with PE after contrast-enhanced chest CT examination, in which 74 patients were symptomatic and 105 patients had no symptom. Among asymptomatic 105 patients, 71 patients got CT scans for other reasons including cancer screening and searching infection focus, and 34 patients got CT scans for searching PE due to either apparent or suspicious DVT. The rate of discovering PE was significantly greater in women (0.46%, 90/19,409) than men (0.29%, 89/31,212) (*P* = 0.008). Of the 179 patients with PE, 164 (92%) had some type of risk factor. For both men and women, the most frequent risk factor was a malignant tumor, followed by obesity, long-term bed rest and infection for men and long-term bed rest, obesity and infection for women. The most common malignant tumor was lung cancer. Although taking antipsychotic agent is not advocated as a risk factor, there is a possibility of involvement.

**Conclusions:**

The risk factors for PE were identified in this single-center, retrospective study.

## Background

The mortality of patients who develop acute pulmonary embolism (PE) is high, at 4.1–14.5% [1–3], indicating the seriousness of the condition. The mechanism of onset is as follows: first, the free-floating blood clots formed in the deep vein or right atrium rapidly occlude the pulmonary artery, and over 90% of the cases are caused by deep vein thrombosis (DVT) formed in the lower limbs or pelvis [4, 5]. Factors that cause venous thromboembolism (VTE), as proposed by Virchow in 1856 are (1) blood flow stagnation, (2) vascular endothelial disorder, and (3) hypercoagulability [6]. Numerous underlying conditions affect the onset of PE, and PE occurs in patients admitted to various departments. However, most reports of PE in Japan include cases from only a single department. This study aimed to investigate the background of patients who presented with PE on contrast-enhanced chest computed tomography (CT) and to explore the risk factors for PE.

## Methods

### Patients

This study included all 50,621 patients who were admitted to the Tsukuba Medical Center Hospital between January 1, 2013 and December 31, 2017. Because we used the records of hospitalized patients, same patients who admitted to hospital another period were counted separately.

### Diagnosis of PE

PE was diagnosed after contrast-enhanced chest CT examination. There was no algorithm for diagnosing PE in our hospital. The condition of CT scanning was not unified, but there was possibility of PE or DVT, we used specific condition CT to find a VTE. After injection of a contrast medium, we scanned chest 15–20 s later and lower limbs 3 min later.

### Examination medical records

A retrospective examination of medical records was conducted to extract data on sex, age, risk factors related to blood flow stagnation (obesity, long-term bed rest, cardiopulmonary disease, cast fixation, long-term sitting), risk factors related to vascular endothelial disorder (surgery, trauma/fracture, central venous catheterization, catheter tests/treatments, vasculitis, antiphospholipid antibody syndrome, history of VTE), and risk factors related to hypercoagulability (malignant tumor, use of oral contraceptives/low-dose estrogen progestin/steroids, infection, inflammatory enteric disease, polycythemia, protein C or protein S deficiency, dehydration). For patients with multiple risk factors, each factor was counted separately for the analysis. For patients with a malignant tumor, the frequency of PE was calculated by cancer type. The χ^2^ test was used for analysis, and *P* < 0.05 was considered significant.

### Summary of facilities

Our hospital is a 453-bed community hospital primarily equipped to handle emergency medicine. Emergency admission to the cardiology and neurology departments is relatively common, and perinatal conditions are not handled. This study was approved by the institutional review board at Tsukuba Medical Center Hospital.

## Results

### Patients’ profile: symptomatic and asymptomatic, association with DVT

**Fig. 1:**
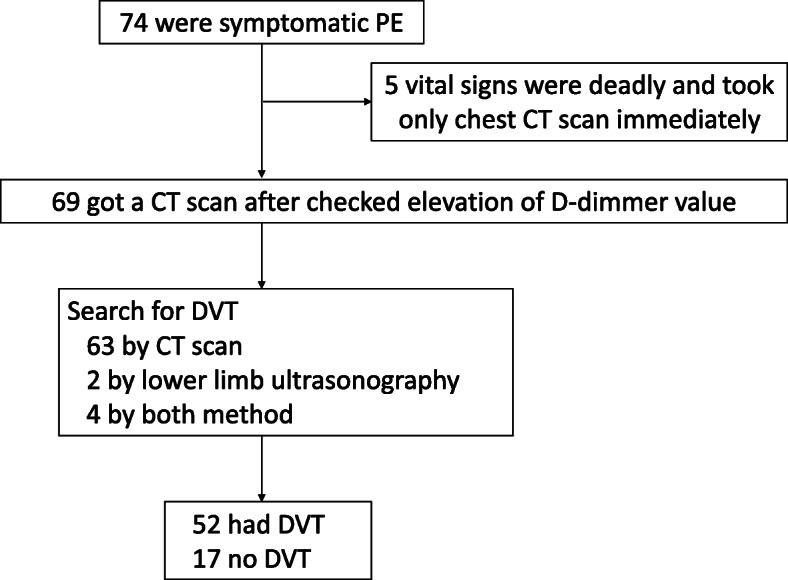
Screening method of symptomatic PE patients

**Fig. 2:**
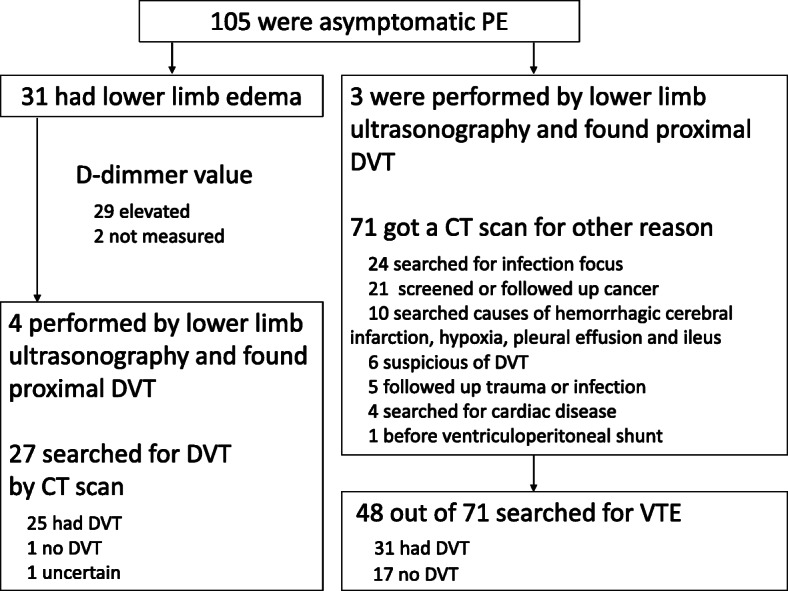
Screening method of symptomatic PE patients

Of all inpatients, 179(0.35%) out of 50,621 were diagnosed with PE after contrast-enhanced chest CT examination, in which 74 patients were symptomatic and 105 patients had no symptom. Among asymptomatic 105 patients, 71 patients got CT scans for other reasons including cancer screening and searching infection focus, and 34 patients got CT scans for searching PE due to either apparent or suspicious of DVT. 74 patients had symptoms such as dyspnea, chest pain, fever, fainting, cough, wheezing, hemoptysis and palpitations [7]. 74 symptomatic patients and 34 asymptomatic patients (31 had lower limb edema and 3 had proximal DVT) were made CT scanning for a suspicious of PE. 5 symptomatic patient’s vital signs were deadly, took only chest CT scan immediately. In asymptomatic patients, 4 patients with lower limb edema and 3 patients with elevated D-dimmer value were made lower limb ultrasonography prior to CT scan and had proximal DVT. Left 27 patients with lower limb edema searched for DVT by CT scan, 25 patients had DVT. 71 patients got a CT scan for other reasons and diagnosed PE coincidentally. 48 out of 71 patients searched for DVT and 31 had DVT. (Figs. 1,2).

PE was more proximal pulmonary artery than subregion branch in all patients. However, there was possibility of overlooking peripheral PE because it was not routine work to search the PE in asymptomatic patient.

### Gender and age

The rate of discovering PE was significantly greater in women (0.46%, 90/19,409) than men (0.29%, 89/31,212) (*P* = 0.008). Table 1 show the number of patients with PE by sex and age group.

**Table 1:** Distribution of the number of patients by age and sex

Age(y)	Male(%)	Female(%)
10–19	1 (1)	0 (0)
20–29	1 (1)	1 (1)
30–39	8 (9)	2 (2)
40–49	13 (15)	7 (8)
50–59	14 (16)	11 (12)
60–69	18 (20)	15 (17)
70–79	17 (19)	28 (31)
80–89	13 (15)	22 (24)
90–99	4 (4)	4 (4)

In men, the number of patients increased gradually from the 30s age group and reached a peak around the 60–70s age group. In contrast, in women, the number increased from the 40s age group, with a relatively steep peak at the 70–80s age group. In men, the rate of discovering PE was not different between those under age 60 years (0.32%, 37/11,609) and those 60 years and older (0.27%, 52/19,603) (*P* = 0.3). In contrast, in women, the rate was significantly greater in those 60 years and older (0.63%, 69/11,009) than in those under 60 years of age (0.25%, 21/8400) (*P* = 0.0001).

### Risk factors for DVT/PE

Of the 179 patients with PE, 164 (92%) had some type of risk factor (Table 2).

**Table 2:** The risk factors of patients with PE separated by gender

	Risk factor	Male(%)	Female(%)	*P*-value
Blood flow stagnation	Obesity	20 (15)	17 (13)	0.63
	Long-term bed rest	18 (13)	23 (17)	0.37
	Sitting for a long time	3 (2)	4 (3)	0.69
	Cardiopulmonary disease	4 (3)	7 (5)	0.34
	Trauma, Fracture, Cast fixed	11 (8)	8 (6)	0.50
	Central venous catheter	0 (0)	1 (1)	0.31
	Surgery	9 (7)	9 (7)	0.97
Vascular endothelial disorder	Antiphospholipid antibody syndrome and collagen disease	6 (4)	2 (1)	0.16
	Past medical history of VTE	2 (1)	1 (1)	0.57
	Cancer	24 (18)	23 (17)	0.92
Hypercoagulability	OCs, LEP, Steroids	2 (1)	5 (4)	0.24
	Infection	18 (13)	17 (13)	0.89
	Inflammatory enteric disease	1 (1)	2 (1)	0.55
	Polycythemia	1 (1)	1 (1)	0.99
	Protein C or Protein S deficiency	2 (1)	0 (0)	0.16
	Dehydration	4 (3)	6 (5)	0.50
	No risk factor	9 (7)	6 (5)	0.44

There was difference in the frequency of CT scanning between cancer and other diseases. 1 patient with colon cancer or lung cancer got a CT scan every 3 months, 1 patient with lung cancer got a CT scan every 6 months after the treatment. 8 cancer patients (6 patients with lung cancer, 1 patient with colon cancer, 1 patient with breast cancer) who were under the treatment got a CT scan every time judging the therapeutic effect. Other cancer patients got a CT scan one time only to investigate the causes of symptoms. Other than cancer patients, 17 patients got a second time CT scan, because 6 patients had hypoxia, 6 patients suspected DVT, 5 patients followed up trauma or infection. Other patients got a CT scan one time only.

We used Wells score and defined that long-term bed rest was more than 72 h [8] and sitting for a long time was more than 5 h [9]. 22 out of 41 patients, long-term bed rest was in-hospital outbreak. They were forced long-term bed rest due to consciousness or trauma.

Regarding to surgery, trauma/fracture and central venous catheterization, refer to Wells score, we determined that the time was less than 3 months before admission. The median time to onset, 10 (0–60) days at operation, 10 (0–72) days at trauma, fracture and cast fixed, and 8 days at central venous catheter. 1 patient after operation of osteoarthritis of the knee had 60 days, 2 patients after fractured had 17 days or 60 days before administration, other patients were admitted immediately after injured or already in the hospital to get surgery.

Only one patient who had past medical history of VTE took anticoagulant therapy, warfarin, other patients were self-interruption or not prescribed.

Patients for unknown reason were measured thrombus predisposition (Protein C or S, anti-cardiolipin antibody and lupus anti-core grant), and 2 patients were diagnosed protein C or S deficiency, 1 patient was diagnosed antiphospholipid antibody syndrome.

The risk factor for the development of PE could not be identified in 15 patients.

Although taking antipsychotic agent is not advocated as a risk factor, 9 patients with schizophrenia were taking antipsychotic agent.

### Gender difference

Although there were no significant differences between men and women in the percentage of patients with each PE risk factor, long-term bed rest was more common in women, at 17%, compared with men, at 13%, and trauma/fracture/cast fixation was more common in men, at 8%, than in women, at 6%. A total of 33(89%) obese patients had another risk factor. For both men and women, the most frequent risk factor was a malignant tumor, followed by long-term bed rest and infection.

### Contribution of cancer-bearing

47 patients with PE and a malignant tumor. 35 patients had advanced cancer. 27 patients were diagnosed cancer after PE diagnosis. Seventeen patients were under the treatment (15 patients had chemotherapy, 1 patient had hormone therapy, 1 patient had surgery). Ten out of 17 patients who were under the treatment got a CT scan to judge therapeutic effect, 7 patients were PD, 2 patients were PR, 1 patient was SD. 3 patients got a CT scan regularly to follow up after treatment and 1 patient had seen recurrence of cancer. 45 patients (95%) had active cancer.

Patients with lung cancer were the most common, accounting for 34% of the 47 patients. The rate of discovering PE by cancer type was greatest in ovarian cancer, at 3.3%, followed by endometrial cancer, at 2.4% (Table 3).

**Table 3:** Numbers and incidence of patients with PE by each cancer type

Cancer type	Number of hospitalized patients	Number of patients with PE(%)
Ovarian cancer	152	5 (3.3)
Endometrial cancer	122	3 (2.4)
Gallbladder cancer	89	2 (2.2)
Lung cancer	1258	16 (1.3)
Colon cancer	1177	9 (0.8)
Gastric cancer	693	3 (0.4)
Breast cancer	1109	5 (0.5)
Prostate cancer	881	3 (0.3)
Kidney cancer	816	1 (0.1)
Cervical cancer	287	0 (0)

The rate of discovering PE in ovarian cancer was not significantly different from that of endometrial cancer, gallbladder cancer, and lung cancer, but it was significantly greater compared with that of colon cancer, stomach cancer, breast cancer, prostate cancer, kidney cancer, and cervical cancer (*P* = 0.0001 to 0.01).

## Discussion

Although there are conflicting reports that the incidence of PE is higher in men [10, 11] or is not different between men and women [12], the incidence is 1.5-fold higher in Japanese women than men, peaking in the 60–70s age group [13]. The present results also showed that the discovery rate was greater in women and peaked around the 60–70s, corroborating previous reports from Japan. PE is thought to more likely occur in older individuals than in younger individuals because the morbidity of underlying conditions, such as cerebrovascular disease/neurological disease, long-term bed rest from a lumbar compression fracture, malignant tumor, and bacterial infections including aspiration pneumonia/pyelonephritis, that are likely to cause VTE increases with increasing age. In particular, in postmenopausal women, events such as cardiovascular diseases and fractures increase rapidly, and this is considered to be the reason why PE is frequently observed in older women.

In PE patients with a malignant tumor, lung cancer was the most common tumor. This is because patients with lung cancer are the most common cancer inpatients at our hospital, and because smoking, a risk factor for lung cancer [14], is also a risk factor for VTE [15]. Thus, this was an expected outcome. Analysis of the PE discovery rate by cancer type showed that patients with ovarian cancer had the highest rate. Before treatment, PE was discovered in 8.8–13.3% of ovarian cancer [16, 17], 3.0–4.7% of endometrial cancer [16, 18], 1.1–1.4% of cervical cancer [16, 19], and 2.9% of advanced pancreatic cancer [20] cases. Moreover, whereas PE was not discovered in bladder cancer or stomach cancer cases, DVT was found in 13.9 and 4.4% of the patients, respectively [21]. VTE is common in ovarian cancer due to vascular dehydration caused by ascites [16] and venous compression by a large tumor [22]. In clear cell carcinoma, Factor VII is activated via the extrinsic blood coagulation pathway, leading to the production of tissue factors that augment coagulation [23, 24], and this is thought to be one of the causes for high VTE rates.

Although taking antipsychotic agent was not advocated as a risk factor, there was a possibility of involvement. Antipsychotics are known to induce pulmonary vasospasm and contraction, as well as platelet aggregation via the serotonin-like action of 5HT2/D2 antagonists [25]. Taking antipsychotics for more than 24 months increases the risk for VTE by 32%, and by 73% with atypical antipsychotics [26]. In Japan, the Ministry of Health, Labour and Welfare in 2010 recommended the addition of PE and DVT as serious side effects to the package inserts of antipsychotics such as haloperidol, blonanserin, clozapine, and risperidone. The use of antipsychotics should perhaps be noted as a risk factor for VTE.

## Conclusions

In our hospital, the rate of discovering PE was high in women who were at least 60 years old. In 92% of the cases, some type of risk factor for onset was identified. The most frequently observed risk factor was a malignant tumor. Lung cancer was the most common by the number of patients, and ovarian cancer was the highest by frequency of discovery. Other risk factors were infection, long-term bed rest and obesity. 89% of obese patients had another risk factor. Antipsychotic drugs may have been associated with PE in some patients.

There are some limitations of this study. Firstly, the sample size is small. 179 patients were selected, but the number of patients would not be sufficient to collect relevant date. Secondly, patients had some risk factors, so we couldn’t declare the most influence risk factors. Finally, there was limitation of department in our hospital, therefore there was the possibility of overlooking the risk factor or undiagnosed diseases. Furthermore, we were unable to fully analyze the date of patients who had not PE, because some medical records were disposed, so we couldn’t do multivariate analysis.

Though there are limitations with this single-center retrospective study, it demonstrated the risk factors for PE in patients who presented with PE.

## Data Availability

The datasets used and/or analyzed during the current study are available from the corresponding author on reasonable request.

## Abbreviations

PE: Pulmonary embolism
CT: Computed tomography
VTE: Venous thromboembolism
DVT: Deep vein thrombosis
BMI: Body mass index
OCs: Oral contraceptives
LEP: Low dose Estrogen Progestin
RECIST: Response evaluation criteria in solid tumors
NED: No evidence of disease
Rec: Recurrence
PD: Progressive disease
SD: Stable disease
PR: Partial response
